# Nationwide Analysis of Palliative Care Utilization Among Ischemic Stroke Hospitalizations

**DOI:** 10.3390/healthcare14193194

**Published:** 2026-09-28

**Authors:** Muhammad Roshan Asghar, Ali Al-Salahat, Alexander Hall, Nicole Horio, Nirmala Persaud, Ripudaman Kahlon, Jagkirat Singh, Rohan Sharma

**Affiliations:** 1 Neurology Department, Creighton University, Omaha, NE 68124, USA; muhammadroshanasghar@creighton.edu (M.R.A.); ripukahlon@creighton.edu (R.K.); jagkiratsingh@creighton.edu (J.S.); rohansharma@creighton.edu (R.S.); 2 Department of Clinical Research and Public Health, School of Medicine, Creighton University, Omaha, NE 68124, USA; alexanderhall@creighton.edu; 3 School of Medicine, Creighton University, Omaha, NE 68124, USA; nicolehorio@creighton.edu (N.H.); nirmalapersaud@creighton.edu (N.P.)

**Keywords:** palliative care, ischemic stroke, disparities, national inpatient sample, end-of-life care, race, socioeconomic status, NIHSS, reperfusion therapy

## Abstract

**Highlights:**

**What are the main findings?**

- Palliative care (PC) utilization among ischemic stroke hospitalizations ranged between 5.34% in 2016 and 6.40% in 2022, remaining low overall, with hospitalizations involving Black and Hispanic patients having significantly lower odds of PC receipt compared with those involving White patients.
- In adjusted models, higher NIHSS scores, malignant neoplasm, respiratory failure, and cerebral edema demonstrated the strongest association with PC receipt among clinical variables, while receipt of reperfusion therapies was inversely associated with PC use.

**What are the implications of the main findings?**

- System-level interventions, including culturally tailored outreach, workforce development, and tele-palliative care, are needed to reduce persistent racial/ethnic, socioeconomic, and institutional disparities in palliative care access for ischemic stroke patients.
- Palliative care should be integrated across all stages of ischemic stroke care, including after reperfusion therapies, consistent with the 2025 American Heart Association scientific statement.

**Abstract:**

**Background/Objectives**: Palliative care (PC) is an integral component of ischemic stroke management, yet contemporary nationwide data on its utilization, recent utilization rates, and associated disparities are lacking. This study examined PC utilization rates, demographic disparities, and clinical factors associated with receipt of PC (PC) across ischemic stroke hospitalizations in the United States (US) from 2016 to 2022. **Methods**: This retrospective cross-sectional study used data extracted from the National Inpatient Sample (NIS) to identify adult hospitalizations primary for ischemic stroke, identified via validated International Classification of Disease—10th Clinical Modification (ICD-10-CM) codes. PC use was identified using the code Z51.5. Multivariable logistic regression was performed to identify factors independently associated with PC, adjusting for demographics, hospital characteristics, clinical severity (National Institutes of Health Stroke Scale [NIHSS] category, Charlson Comorbidity Index), complications, and receipt of thrombolysis/thrombectomy. **Results**: Among 4350,924 weighted ischemic stroke hospitalizations, 253,555 (5.83%) received PC, ranging from 5.34% in 2016 to 6.40% in 2022. Hospitalizations involving Black (adjusted odds ratio [aOR], 0.71; 95% CI, 0.69–0.74) and Hispanic (aOR, 0.73; 95% CI, 0.70–0.77) individuals had significantly lower odds of PC receipt compared with those involving White individuals. Lower-income quartiles, rural hospitals, and non-teaching hospitals were independently associated with reduced odds of PC receipt. Compared with NIHSS scores of 0–9, higher NIHSS scores demonstrated a graded association with PC use (NIHSS 20–29: aOR, 8.53; NIHSS 30–39: aOR, 12.22). Malignant neoplasm (aOR, 3.83), respiratory failure (aOR, 3.04), and cerebral edema (aOR, 2.82) showed clinically large statistically significant associations with PC. Receipt of reperfusion therapies were inversely associated with PC use (aOR, 0.85 for thrombolysis; aOR, 0.71 for thrombectomy). **Conclusions**: PC utilization among ischemic stroke hospitalizations remains low, with persistent racial/ethnic, socioeconomic, and institutional disparities. System-level interventions are needed to expand equitable PC access across all stages of ischemic stroke care, including patients who receive reperfusion therapies.

## 1. Introduction

Stroke remains a leading cause of death and long-term morbidity in the United States (US), with a mortality rate of 39.0 per 100,000 people in 2023 and more than 795,000 new cases annually [1,2]. Advances in acute reperfusion therapies have reduced mortality and morbidity, and consequentially the number of stroke survivors is increasing. Indeed, stroke survivors face long-term cognitive, physical and psychosocial challenges [1]. Palliative care (PC) is emerging as a critical component of comprehensive ischemic stroke management. It operates through an integrated, interdisciplinary framework that addresses symptom burden, aligns treatment decisions with patient and family goals, and facilitates timely transition between curative and comfort-focused care. By targeting the physical, psychological, social, and spiritual domains, PC moderates the trajectory of illness-related suffering and shapes key clinical outcomes, including functional decline, care setting transitions, and end-of-life decision-making. There are multi-dimensional requirements of patients and families throughout the course of the illness. Evidence demonstrates that early PC integration improves quality of life and depression, and may prolong survival [3,4]. Building on this evidence, the 2025 American Heart Association (AHA) statement affirms that PC should be integrated into ischemic stroke care at all stages, not solely at end-of-life [2]. Identifying factors associated with PC is therefore essential to understanding when and for whom these interventions are mostly utilized. Both acute and chronic needs of patients with ischemic stroke can be addressed by PC, including decision-making under time pressure, and adaptation to disability [4].

Despite clear endorsements of the importance of PC by the AHA and the Neurocritical Care Society, PC has been under-utilized in stroke care. A Prior National Inpatient Sample (NIS) study based on data from 2010 to 2012 showed that PC was used in only 6.2% of stroke hospitalizations, with significant demographic disparities [5]. Another recent multicenter cohort study showed that PC was used in 20% of severe ischemic stroke patients, with wide institutional variation ranging from 11.9% to 43% [6]. Additionally, significant racial/ethnic disparities in the use of PC after stroke were highlighted in a prior 2007–2011 NIS study [7]. However, recent data on the consultation rates, disparities and clinical factors associated with PC utilization in ischemic stroke is lacking. Revisiting and addressing these vital questions and providing a recent nationwide update is a timely need. This study aimed to evaluate recent utilization rates, demographic disparities and clinical factors associated with PC among ischemic stroke hospitalizations in the United States (US) from the NIS (2016–2022), providing a contemporary and comprehensive analysis.

## 2. Materials and Methods

### 2.1. Study Design, Database and Population

We followed the RECORD extension of the STROBE reporting guidelines. Data for this retrospective study were abstracted from the 2016–2022 National Inpatient Sample (NIS). The NIS is the largest publicly available all-payer inpatient healthcare database in the US, part of the Healthcare Cost and Utilization Project (HCUP) and sponsored by the Agency for Healthcare Research and Quality (AHRQ) [7]. The NIS approximates a 20% stratified sample of all discharges from US hospitals. When weighted, the NIS represents approximately 35 million hospitalizations annually and an estimated 241 million discharges over the study period. The NIS is a publicly available, fully de-identified, Health Insurance Portability and Accountability Act (HIPAA)-compliant database.

The study population consisted of all adult hospitalizations (patients aged ≥18 years at admission) with a primary diagnosis of ischemic stroke. Hospitalizations were identified using the International Classification of Disease–10–Clinical Modification codes, consistent with previous work [8].

### 2.2. Outcome Measures, Co-Variates and Stratifications

The primary outcome was receipt of PC during the index hospitalization, identified using the ICD-10-CM encounter code Z51.5. This code captures encounters in which PC services were documented during the hospitalization (consistent with prior studies) [1,5]. Multiple validity studies showed that Z51.5 carried a specificity of 99–100% and sensitivity of up to 80% [9,10,11]. Patient-level variables included age, biological sex, race/ethnicity (White, Black, Hispanic, other), primary payer (Medicare, Medicaid, private insurance, other), and median household income quartile for the patient’s ZIP code (used as a proxy for socioeconomic status). Hospital-level variables included teaching status (urban teaching, urban non-teaching, rural), hospital bed size (small, medium, large), hospital census region (Northeast, Midwest, South, West), and admission type (weekday versus weekend). Hospitalizations missing patient-level variables (which accounted for 4% of the eligible sample) were excluded from adjusted analyses.

Clinical variables included the Charlson Comorbidity Index (CCI), NIHSS score (categorized as 0–9, 10–19, 20–29, 30–39, and 40–42 based on ICD-10-CM codes R29.7xx), and the presence of specific comorbidities and complications identified by ICD-10-CM codes: concomitant non-traumatic intracerebral hemorrhage (ICH), cerebral edema, coma, respiratory failure, sepsis, non-infectious systemic inflammatory response syndrome (SIRS), kidney disorders, aspiration pneumonitis, urinary tract infection (UTI), malignant neoplasm, hematological disorders, and hypertensive crisis. Procedural variables included receipt of intravenous thrombolysis, mechanical thrombectomy, craniectomy, and duration of mechanical ventilation (≤24 h, 24–96 h, >96 h), identified using ICD-10-Procedure codes (Supplementary Table S1). Given that these variables were defined by the presence or absence of a qualifying code, they had no missing values; patients without a code were classified as not having the condition or procedure. The exceptions were NIHSS score and duration of mechanical ventilation, which are available only when coded. For these two variables, we treated each as categorical and added a separate ‘missing’ category for patients without a relevant code.

### 2.3. Statistical Analysis

All analyses incorporated NIS-provided discharge weights, strata, and cluster variables to account for the complex survey design and generate nationally representative estimates. Descriptive statistics for continuous variables were reported as mean and standard deviation, while categorical variables were reported as frequencies and percentages. All baseline demographic and clinical characteristics were compared using standardized mean differences.

Formal assessment of an annual trend in palliative care receipt was tested using a linear orthogonal polynomial contrast on the logit scale. A multivariable logistic regression model was estimated to evaluate differences in the association between patient and clinical characteristics with receipt of PC. Model covariates included all the demographic, hospital, clinical, and procedural variables described above. Before fitting the multivariable model, we examined covariate-specific expected cell counts stratified by PC to identify sparse data, and calculated variance inflation factors (VIFs) to assess multicollinearity; expected counts were large and VIFs were all < 1.6. Results are reported as adjusted odds ratios (aOR) with 95% confidence intervals (CI). Due to the number of variables, both the descriptive tables and the inferential figures are divided into two parts: (1) demographic and hospital characteristic covariates and (2) clinical covariates.

All analyses were conducted using SAS version 9.4 (SAS Institute, Cary, NC, USA), and statistical significance was set at *p* < 0.05 throughout. This study was acknowledged as Not Human Subjects Research by Creighton University’s Institutional Review Board (InfoEd Record Number: 2044754).

## 3. Results

### 3.1. Study Population and Recent Palliative Utilization Rates

A total of 4350,924 weighted hospitalizations (unweighted: 870,185) with a primary discharge diagnosis of ischemic stroke occurred from 2016 to 2022, of which 253,555 (5.83%) received PC (unweighted 50,711). The observed proportion of ischemic stroke hospitalizations with PC over the study period ranged between 5.34% in 2016 and 6.40% in 2022. This linear trend was statistically significant, indicating a 20% relative increase (OR = 1.20, 95% CI [1.14, 1.30], p_trend_ = 0.002).

### 3.2. Demographic Characteristics

Baseline hospitalization characteristics and standardized mean differences (SMD) are presented in Table 1. Adjusted model results are presented in Figure 1. Female sex (aOR, 1.32; 95% CI, 1.29–1.35), increasing age (aOR per year, 1.08; 95% CI, 1.08–1.09), and higher CCI (aOR per unit, 1.13; 95% CI, 1.12–1.14) were independently associated with higher odds of PC receipt. Hospitalizations involving non-Hispanic Black individuals (aOR, 0.71; 95% CI, 0.69–0.74) and Hispanic individuals (aOR, 0.73; 95% CI, 0.70–0.77) had significantly lower odds of PC use compared with those involving White individuals.

Compared with Medicare beneficiaries, hospitalizations with Medicaid (aOR, 1.60; 95% CI, 1.51–1.69), private insurance (aOR, 1.43; 95% CI, 1.37–1.50), and other insurance (aOR, 2.19; 95% CI, 2.06–2.33) had higher odds of PC receipt. Hospitalizations involving patients from the lowest (aOR, 0.91; 95% CI, 0.88–0.95) and second-lowest (aOR, 0.95; 95% CI, 0.91–0.98) income quartiles were less likely to receive PC compared with those from the highest quartile. No significant difference was observed for the third-highest income quartile (aOR, 0.98; 95% CI, 0.95–1.02).

### 3.3. Hospital-Level Characteristics

Hospitalizations at rural hospitals (aOR, 0.93; 95% CI, 0.88–0.98) and urban non-teaching hospitals (aOR, 0.90; 95% CI, 0.86–0.93) had lower odds of PC compared with urban teaching hospitals. Weekend admissions were associated with higher odds of PC (aOR, 1.13; 95% CI, 1.11–1.16). Small-bed hospitals had lower odds of PC compared with large-bed hospitals (aOR, 0.93; 95% CI, 0.90–0.97), while medium-bed hospitals did not differ significantly (aOR, 1.02; 95% CI, 0.98–1.05). Hospitalizations in the Northeast (aOR, 0.88; 95% CI, 0.83–0.92) and South (aOR, 0.91; 95% CI, 0.87–0.95) had lower odds of PC compared with the West, while the Midwest did not differ significantly (aOR, 1.00; 95% CI, 0.96–1.05).

### 3.4. Clinical Factors Associated with Palliative Care Utilization

Baseline clinical factors and associated standardized mean differences (SMD) are presented in Table 2.

Higher NIHSS scores demonstrated a graded increase in the likelihood of PC receipt. Compared with the reference category (NIHSS 0–9), hospitalizations involving patients with NIHSS 10–19 (aOR, 2.27; 95% CI, 2.19–2.35), 20–29 (aOR, 8.53; 95% CI, 8.10–8.98), 30–39 (aOR, 12.22; 95% CI, 11.08–13.48), and 40–42 (aOR, 12.35; 95% CI, 8.00–19.07) had progressively higher odds.

Clinical diagnoses associated with higher odds of PC included malignant neoplasm (aOR, 3.83; 95% CI, 3.58–4.10), respiratory failure (aOR, 3.04; 95% CI, 2.92–3.16), cerebral edema (aOR, 2.82; 95% CI, 2.71–2.94), SIRS (aOR, 2.02; 95% CI, 1.78–2.29), coma (aOR, 1.99; 95% CI, 1.91–2.07), aspiration pneumonitis (aOR, 1.90; 95% CI, 1.82–1.99), concomitant intracerebral hemorrhage (aOR, 1.58; 95% CI, 1.50–1.65), sepsis (aOR, 1.58; 95% CI, 1.48–1.68), UTI (aOR, 1.12; 95% CI, 1.08–1.16), and hematological disorders (aOR, 1.11; 95% CI, 1.08–1.14). Hypertensive crisis was associated with marginally lower odds of PC use (aOR, 0.94; 95% CI, 0.90–0.98).

Receipt of reperfusion therapies was inversely associated with PC after adjustment: thrombolytics (aOR, 0.85; 95% CI, 0.81–0.88) and mechanical thrombectomy (aOR, 0.71; 95% CI, 0.67–0.75). Craniectomy was not significantly associated with PC (aOR, 1.33; 95% CI, 0.81–2.20). Mechanical ventilation of any duration was associated with increased odds of PC: ≤24 h (aOR, 1.75; 95% CI, 1.63–1.88), 24–96 h (aOR, 2.14; 95% CI, 2.02–2.27), and >96 h (aOR, 1.87; 95% CI, 1.75–2.00), as shown in Figure 2.

## 4. Discussion

This study represents the largest and most contemporary national analysis of PC utilization among patients hospitalized with ischemic stroke in the United States, encompassing over 4.3 million weighted hospitalizations from 2016 to 2022. Our findings demonstrated that PC utilization remained low overall and was inequitably distributed across racial, socioeconomic, and institutional lines. These results have important implications for clinical practice, health policy, and the equitable delivery of patient-centered ischemic stroke care.

### 4.1. Observed Rates over Time in Palliative Care Utilization

PC use remained low, ranging from 5.34% in 2016 to 6.4% in 2022. This finding is consistent with prior studies, despite the growing awareness of the importance of PC in acute neurological conditions and professional society endorsements [1,4]. Additionally, the proportion of PC receipt was close to the one found in prior nationwide studies, performed almost a decade ago [7]. This highlights that PC remains under-utilized in ischemic stroke even though its burden has increased. Moreover, the overall rate remains low compared to the substantial morbidity, mortality and burden associated with severe ischemic stroke. Prior studies showed a 20% reported PC use rate in ischemic stroke with an NIHSS of 10 or more [6]. The prior study by Singh et al. reported the PC use rate among all stroke subtypes, while the present study focused exclusively on ischemic stroke. This suggests that the pace of PC adoption has plateaued despite a decade of advocacy. Additionally, Chu et al. analyzed the NIS data from 2006 to 2015 and reported that comfort care intervention use among ischemic stroke patients increased five-fold over the decade, yet the absolute rate remained at 3.8% [12]. This stagnation is concerning given that the AHA 2025 scientific statement emphasized that PC should be integrated into all stroke care stages and not limited to end-of-life scenarios [1]. The multicenter cohort study (2016–2019) that focused on older people with severe ischemic stroke showed that only 20% had received PC, with high institutional variability (11.9% to 43%) [13]. This wide variability suggests that system-level factors, rather than patient-level clinical need alone, could be major determinants of PC delivery. Notably, the minor change in PC use found across our study period could partly be attributed to the influence of the COVID-19 pandemic, which heightened awareness of goals-of-care and palliative care needs across all hospitalized populations [14,15]. Whether this pandemic-related increase in PC awareness will be sustained in the post-pandemic era warrants ongoing surveillance.

### 4.2. Racial and Ethnic Disparities

We found persistent and significant racial/ethnic disparities in PC utilization. Hospitalizations involving non-Hispanic Black and Hispanic individuals had significantly lower odds of PC receipt compared to those involving White individuals, even after adjusting for clinical severity, comorbidities, and hospital-level factors. A prior NIS study found that the odds of PC receipt for both White and minority stroke patients were lower in minority compared with White patients [5]. This suggests that system-level factors could be major contributors to the observed racial/ethnic disparities.

The 2025 AHA statement on palliative care in stroke underscored that inequities by race/ethnicity in advanced stroke care are pervasive and do not reflect patients’ preferences. These disparities are not unique to stroke. In oncology, certain racial/ethnic groups and those with lower socioeconomic status face structural, systemic and interpersonal barriers to PC [16,17]. Additionally, a recent study of Medicare decedents with poor-prognosis cancers found that disparities in PC use remained stable between 2018 and 2023, even though PC delivery increased [18]. Black and Hispanic individuals were also less likely to complete initial PC visits [19]. These disparities have also been found in other medical conditions, including heart failure, renal disease, dementia and serious respiratory illness [20,21,22,23,24,25]. This cross-disease consistency highlights that racial/ethnic disparities in PC utilization may reflect broader systemic inequities in the healthcare system.

### 4.3. Socioeconomic and Insurance-Related Disparities

Hospitalizations involving patients from the lowest and second-lowest income quartiles had significantly lower odds of receiving PC compared with those from the highest income quartile. This gradient in PC utilization is consistent with broader evidence demonstrating that low socioeconomic position is associated with not receiving specialist PC across serious illnesses. A systematic review and meta-analysis found that people living in the most deprived neighborhoods were more likely to not receive specialist PC and to die in hospital rather than at home, with evidence of a dose–response relationship across the social stratum [26].

The finding that hospitalizations associated with Medicaid, private insurance, and other insurance had higher odds of PC use compared with Medicare is noteworthy and may appear counterintuitive. However, this may reflect unmeasured/residual confounding by age and clinical context: Medicare beneficiaries constitute most ischemic stroke hospitalizations and include a broad spectrum of stroke severity, whereas non-Medicare patients who receive PC may represent a more clinically selected population with higher acuity or specific clinical circumstances prompting PC utilization.

### 4.4. Sex-Based Differences

Female sex was independently associated with higher odds of PC receipt. This is consistent with some prior studies and opposite to others [7,12,13]. This discrepancy could reflect differences in administrative database studies and chart-review based cohort studies or institutional variation in PC practices. Female individuals affected by ischemic stroke are of older age, with higher rates of comorbidity; in addition, they may have advance care planning and treatment differences. We hypothesize that these factors may explain the higher PC utilization in females. Future studies are needed to understand the underlying reasons driving these differences.

### 4.5. Clinical Factors

We found a clear graded association between NIHSS scores and PC utilization. This severity-dependent pattern is clinically expected and consistent with previous studies focusing on severe ischemic stroke in older people [13]. Another study similarly found that severe stroke measured by NIHSS, insular cortex infarction and cerebral edema with herniation were associated with transition to comfort measures [27]. Our study extends these findings by demonstrating this gradient at a national level across the NIHSS spectrum. It should be noted again that the AHA statement emphasized the importance of incorporating specialized PC across the entire severity spectrum of stroke. People affected by mild and moderate stroke face significant cognitive, physical, and psychosocial challenges that warrant PC evaluation and assessment.

Additional clinical factors associated with significantly higher odds of PC utilization included malignant neoplasm, respiratory failure, mechanical ventilation (of any duration), cerebral edema, SIRS, coma and aspiration pneumonitis. These associations may reflect the role of PC in managing complex and life-threatening complications and facilitating goals-of-care discussions when prognosis is poor.

The inverse association between receipt of reperfusion therapies and PC receipt is an important finding. We hypothesize that this finding could potentially reflect a clinical paradigm in which reperfusion therapy is perceived as having curative intent, potentially delaying or precluding PC discussions. In a decade-long analysis between 2006 and 2015, thrombolysis and endovascular thrombectomy were associated with 6% and 10% greater likelihood of comfort care intervention receipt [12]. The study periods were markedly different between the present study and the aforementioned one. Thrombectomy has become widely available and outcomes have substantially improved [28,29]. The receipt of active and effective treatment should not preclude the integration of PC into ischemic stroke care, especially in the early post-intervention period with prognostic uncertainty [2,30]. However, it is also possible that treatment selection, prognosis, survival, institutional practices and residual confounding may contribute to the association we observed.

### 4.6. Barriers to Palliative Care Integration

Several barriers to PC incorporation in ischemic stroke care need to be addressed in the setting of our findings. In a Canadian stroke survey of physicians, PC services were utilized “sometimes” and more for end-of-life care than for prognostication; prognostic uncertainty was found to be a key barrier to early integration of PC in stroke care [31]. Other studies identified additional barriers, including conflation of PC with end-of-life care or “giving up”, gaps in PC education for neurologists, varying disease trajectories that complicate hospice referral, and workforce shortages of PC specialists [32]. These barriers likely contribute to the low PC use rates observed in our study and underscore the need for educational initiatives, institutional culture changes and workforce development. Tele-palliative or tele-neuro-palliative specialist care might also be a useful tool to mitigate disparities in PC receipt [33,34].

### 4.7. Limitations

This study has several limitations related to the use of administrative databases. First, the NIS relies on ICD-10-CM coding, and the Z51.5 code for palliative care encounters may underestimate true PC utilization, as informal palliative care discussions or primary palliative care provided by the treating team without formal consultation may not be captured. The ICD code, Z51.5, carries very high specificity but relatively lower sensitivity. Second, the NIS does not provide information on the timing, content, or quality of PC, nor does it distinguish between specialist palliative care consultation and primary palliative care. Third, the cross-sectional design precludes causal inference, and observed associations may be influenced by unmeasured confounders, including patient and family preferences, advance directive status, and institutional palliative care availability. Fourth, the NIS does not capture outpatient or post-discharge palliative care utilization, limiting the analysis to inpatient encounters. Sixth, race and ethnicity data in the NIS are derived from hospital records and may be subject to misclassification. Finally, the study period includes the COVID-19 pandemic years (2020–2022), which may have influenced both stroke care patterns and palliative care utilization in ways that are difficult to fully account for. Additional limitations include under-coding of NIHSS scores, hospitalization-level data, the lack of temporal association between PC use and associated factors, and residual confounding.

## 5. Conclusions

This nationwide analysis of over 4.3 million ischemic stroke hospitalizations from 2016 to 2022 demonstrates that PC utilization remains low overall, with significant and persistent disparities based on race/ethnicity, socioeconomic status, hospital factors, and geographic region. Clinical severity, as measured by NIHSS score and the presence of serious co-existent conditions or clinical factors, was highly associated with PC utilization. Hospitalizations involving racial/ethnic minorities, lower-income quartiles, and rural or non-teaching hospitals were significantly associated with lower odds of PC receipt. These findings underscore the urgent need for system-level interventions to expand palliative care access, reduce disparities, and integrate palliative care as a routine component of ischemic stroke care at all stages, consistent with the 2025 AHA scientific statement. Future efforts should focus on culturally tailored interventions, workforce development, institutional culture change, and implementing tele-palliative care and policy reforms to ensure equitable access to high-quality PC for all patients with ischemic stroke.

## Figures and Tables

**Figure 1:**
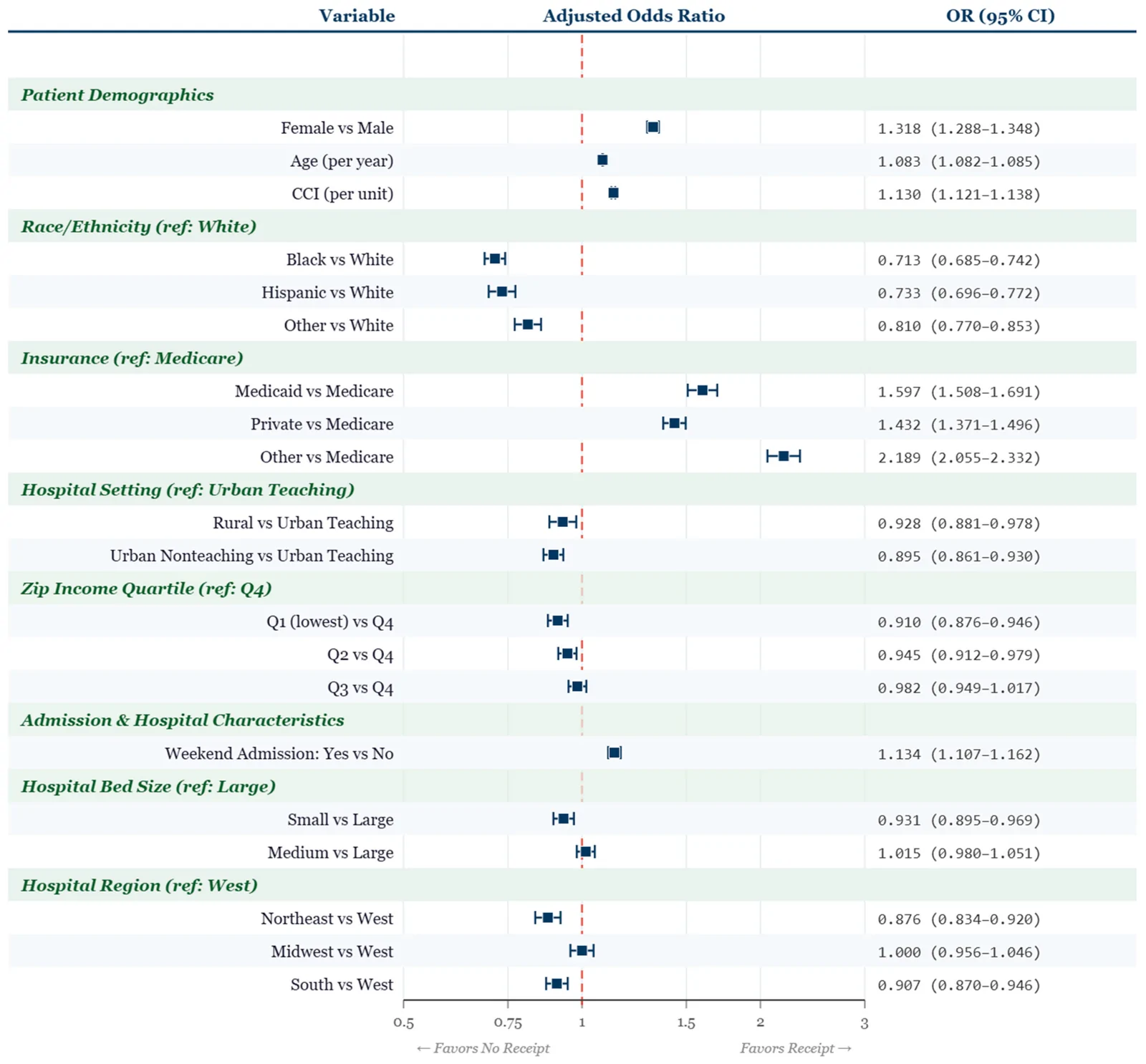
Demographic factors associated with palliative care receipt. Odds ratios represent survey-weighted adjusted odds ratios. Multivariable model adjusted for the all the demographic and clinical factors reported. OR = odds ratio, CCI = Charlson Comorbidity Index, Q = quartile, 95% CI = 95% confidence interval. Red dashed line represents OR of 1. Each square represents the adjusted odds ratio (OR) for the corresponding variable, and the horizontal lines intersecting the squares represent the 95% confidence intervals.

**Figure 2:**
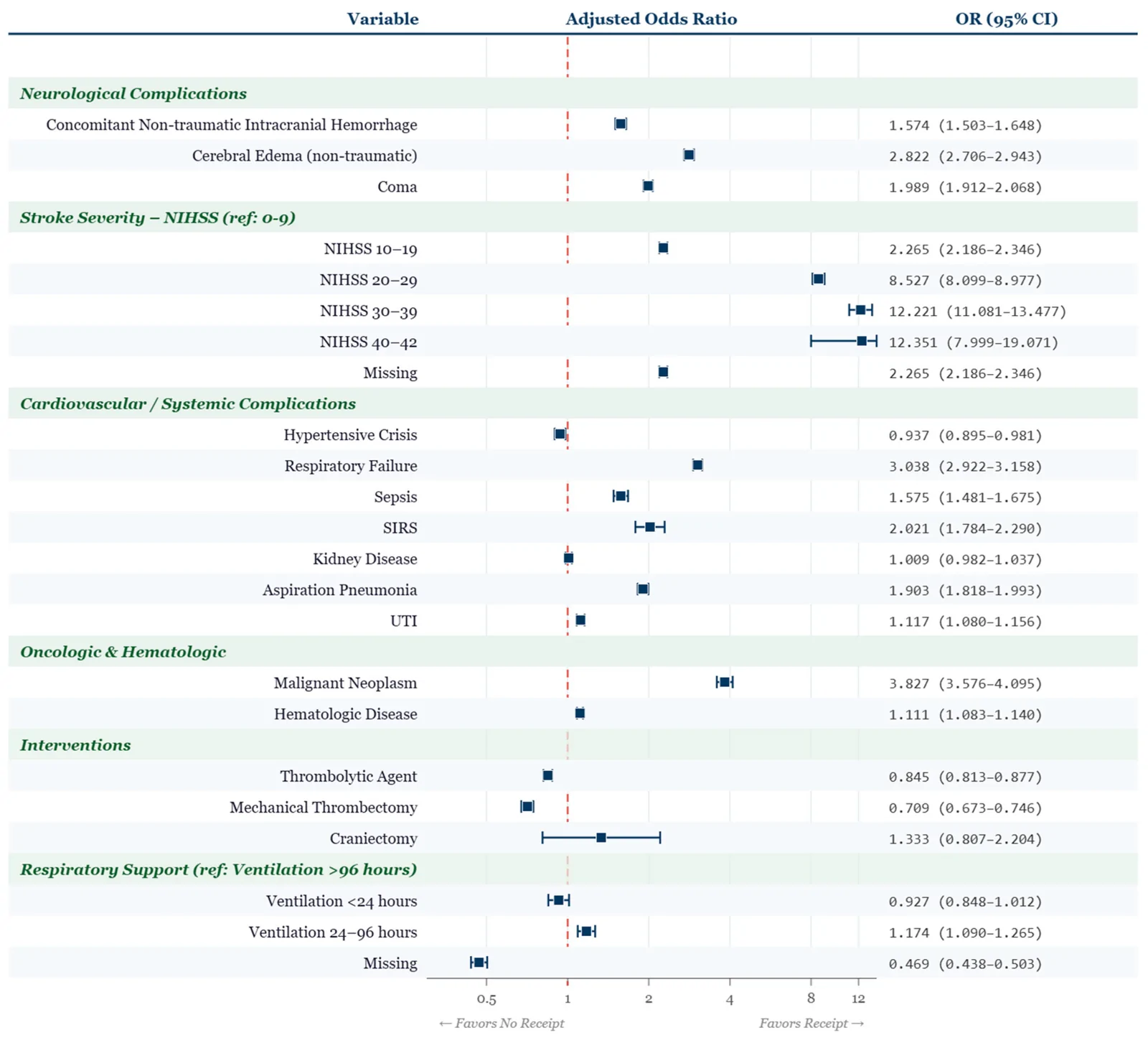
Clinical factors associated with palliative care receipt. Odds ratios represent survey-weighted adjusted odds ratios. Where not stated, reference is absence of disease (i.e., coma has 2.010 odds (higher) of palliative care receipt compared with no coma). Multivariable model adjusted for the all the demographic and clinical factors reported. OR = odds ratio; 95% CI = 95% confidence interval; UTI = urinary tract infection; NIHSS = National Institute of Health Stroke Scale; SIRS = systemic inflammatory response syndrome. Dashed red line represents OR of 1. Each square represents the adjusted odds ratio (OR) for the corresponding variable, and the horizontal lines intersecting the squares represent the 95% confidence intervals.

**Table 1:** Demographic characteristics of ischemic stroke hospitalizations stratified by palliative care receipt from the national-inpatient sample (2016–2022). Counts represent weighted estimates.

	No-Palliative	Palliative	SMD
Hospitalization Count			
Unweighted	819,474	50,711	
Weighted	4,097,369	253,555	
Age	69.68 (13.20)	78.65 (11.70)	0.72
Charlson Comorbidity Index	2.15 (1.71)	3.93 (2.15)	0.47
Female	47.50%	58.67%	0.23
Race/Ethnicity			
White	69.67%	75.94%	0.14
Non-Hispanic Black	16.06%	11.20%	−0.14
Hispanic	8.11%	6.59%	−0.06
Other	5.86%	6.27%	0.02
Insurance			
Medicare	65.04%	78.76%	0.31
Medicaid	9.27%	5.11%	−0.16
Private	19.31%	10.90%	−0.24
Other	6.38%	5.23%	−0.05
Location/Teaching Status			
Rural	7.16%	5.87%	−0.05
Urban Nonteaching	19.11%	15.82%	−0.09
Urban Teaching	73.73%	78.30%	0.11
Income Quartile			
1st–25th	30.70%	27.13%	−0.08
26th–50th	26.69%	26.26%	−0.01
51st–75th	23.78%	24.89%	0.03
76th–99th	18.84%	21.73%	0.07
Weekend Admission	22.57%	26.76%	0.10
Facility Bed Size			
Small	18.03%	14.95%	−0.08
Medium	28.99%	27.11%	−0.04
Large	53.07%	57.94%	0.10
Facility Region			
Northeast	17.09%	17.16%	<0.01
Midwest	21.78%	23.56%	0.04
South	43.07%	38.63%	−0.09
West	18.06%	20.65%	0.07

SMD = standardized mean difference.

**Table 2:** Clinical factors associated with palliative care receipt among ischemic stroke hospitalizations from the national inpatient sample (2016–2022).

Characteristic	No-Palliative	Palliative	SMD
Concomitant non-traumatic intracerebral hemorrhage	3.33%	12.17%	0.33
Cerebral edema (non-traumatic)	3.85%	19.40%	0.50
Coma	7.89%	19.65%	0.35
Coma upon arrival to ER	0.09%	1.26%	0.14
Coma in the field by EMS	0.01%	0.13%	0.04
Coma upon admission	0.10%	1.35%	0.15
Coma 24 h or more after admission	0.07%	1.40%	0.16
Thrombolytic agent	10.34%	13.00%	0.08
Mechanical thrombectomy	3.50%	9.89%	0.26
Craniectomy	0.02%	0.07%	0.03
Hypertensive crisis	7.37%	6.56%	−0.03
NIHSS 0–9	32.33%	12.02%	−0.50
NIHSS 10–19	5.89%	14.17%	0.28
NIHSS 20–29	2.05%	16.15%	0.51
NIHSS 30–39	0.25%	3.72%	0.25
NIHSS 40–42	0.01%	0.04%	0.05
NIHSS not documented	59.98%	55.51%	−0.09
Respiratory failure	5.65%	34.99%	0.78
Sepsis	1.37%	7.83%	0.31
SIRS (non-infectious)	0.33%	1.10%	0.09
Renal disorders	24.79%	37.44%	0.28
Aspiration pneumonitis	2.47%	15.08%	0.46
UTI	7.41%	14.22%	0.22
Malignant neoplasms (including secondary)	1.29%	7.04%	0.29
Hematological disorders	20.75%	32.31%	0.26
Respiratory support vent <24 h	0.86%	4.74%	0.24
Respiratory support vent 24–96 h	1.27%	11.07%	0.42
Respiratory support vent >96 h	1.10%	8.93%	0.36
Missing	96.89%	75.91%	−0.64

SMD = standardized mean difference.

## Data Availability

The data used in this study can be made available by the corresponding author upon reasonable request.

## Supplementary Materials

The following supporting information can be downloaded at: https://www.mdpi.com/article/10.3390/healthcare14193194/s1, Supplemental Table S1. Detailed list of ICD-10 codes used in the study.
