# Supplementary material for: Nationwide Analysis of Palliative Care Utilization Among Ischemic Stroke Hospitalizations
Source: Healthcare (Basel). 2026 Sep 28;14(19):3194. doi: 10.3390/healthcare14193194 (PMC13664885; doi:10.3390/healthcare14193194)
Supplement: Supplementary file 1 [file healthcare-14-03194-s001.zip › healthcare-4503134-supplementary.pdf]

Supplemental Table S1. Detailed list of ICD-10 codes used in the study.

Variable / Description	ICD-10 Code(s)
Concomitant non-traumatic intracerebral hemorrhage	I61
Cerebral edema (non-traumatic)	G93.6
Coma	R40.2
Coma upon arrival to ER	R40.2112
Coma in the field by EMS	R40.2111
Coma upon admission	R40.2113
Coma 24 hours or more after admission	R40.2114
Thrombolytic agent	3E03317, 3E03017, Z92.82
Mechanical thrombectomy	03CG0ZZ, 03CG3Z7, 03CG3ZZ, 03CG4ZZ, 05CL3ZZ, 05CL0ZZ, 05CL4ZZ
Craniectomy	0N500ZZ, 0N503ZZ, 0N504ZZ, 0NB00ZZ, 0NB03ZZ, 0NB04ZZ, 0NT10ZZ, 0NT30ZZ, 0NT40ZZ, 0NT50ZZ, 0NT60ZZ, 0NT70Z
Hypertensive crisis	I16
NIHSS 0–9	R29.70
NIHSS 10–19	R29.71
NIHSS 20–29	R29.72
NIHSS 30–39	R29.73
NIHSS 40–42	R29.74
Respiratory ventilation <24 hours	5A1935Z
Respiratory ventilation 24–96 hours	5A1945Z
Respiratory ventilation >96 consecutive hours	5A1955Z
Respiratory failure	J96
Reversible cerebral vasoconstriction syndrome (RCVS)	I67.841
Sepsis	A40, A41
SIRS, non-infectious	R65.1
Kidney disorders	N00–N99
Aspiration pneumonitis	J69
Urinary tract infection (UTI)	N39.0
Malignant neoplasms, including secondary	C00–C96
Hematological disorders	D50–D89
